# Predicting immune checkpoint inhibitor response in urothelial carcinoma: another step in personalised medicine?

**DOI:** 10.1038/s41416-019-0684-2

**Published:** 2019-12-20

**Authors:** Günter Niegisch

**Affiliations:** 1 Department of Urology, Medical Faculty, Heinrich-Heine-University, Düsseldorf, Germany; 2 Centre for Integrated Oncology Aachen-Bonn-Cologne-Düsseldorf, Cologne, Germany

**Keywords:** Bladder cancer, Predictive markers

## Abstract

Prediction of treatment response is a crucial issue in individualised treatment for cancer patients. In this context, Nassar and colleagues in the accompanying study published in the *British Journal of Cancer* analysed retrospectively a cohort of 62 metastatic urothelial cancer patients treated with immune checkpoint inhibitors and of whom not only clinical but also genomic characteristics were available. Combining molecular and clinical factors in a multivariable analysis they identified lack of visceral metastases, neutrophil-to-lymphocyte ratio (NLR) <5, and high single nucleotide variant (SNV) count (≥10) as independent predictors of treatment response.

## Main

Prediction of treatment response is a crucial issue when aiming at individualised treatment for cancer patients. It is not only important for comparing the efficacy of different treatments but also for weighing their risks and benefits in individual patients with various co-morbidities and prognoses.

In invasive urothelial carcinoma (UC) as well, reliable prediction of response to systemic therapy would be highly valuable to tailor individual treatment approaches. In this regard, the definition of molecular subtypes of UC may pave the way for prediction of response towards conventional chemotherapy or the use of FGFR (fibroblast growth factor receptor) inhibitors in specific patient subgroups.^1–3^ However, response prediction towards immune checkpoint inhibitors (ICI) is rather difficult and adequate biomarkers are lacking.^4^ This is a particular vexing dilemma, since in the 20–30% of patients responding to ICI durable cancer control may be achieved, but potentially more efficient chemotherapy may be withheld in the majority of patients that do not benefit from ICI.^5^ Even worse, some non-responders may experience/suffer deterioration by hyper-progressive disease.^6^

In this context, Nassar et al.^7^ present their data on both clinical and genomic parameters to predict response towards ICI in UC. They analysed retrospectively a cohort of 62 metastatic UC patients treated with ICI in whom not only clinical, but also genomic characteristics (by targeted gene sequencing using an institutional customised gene panel^8^) had been assessed. In brief, single nucleotide variant (SNV) count, an APOBEC mutagenic signature, copy number variant (CNV) count including homozygous *CDKN2B* deletions and enrichment for mutations in certain DNA damage response (DDR) pathways (e.g. homologous recombination and nucleotide excision repair pathways) correlated with response to ICIs in univariate analysis. Apart from these genomic factors, the presence of liver metastases, a high neutrophil-to-lymphocyte ratio (NLR), low haemoglobin and low ECOG PS (≥1) predicted poor response towards ICI treatment. In a multivariable analysis combining molecular and clinical factors, lack of visceral metastases, lower NLR < 5, and high SNV count (≥10) were independent predictors of treatment response. Of note, neither any clinical nor genomic factors were associated with treatment response in a group of 39 patients treated with taxanes instead of ICI. Independent of treatment, the authors observed that survival of patients was correlated with clinical and molecular factors, which included those associated with response to ICI treatment. In this context, presence of visceral metastasis, platelet count and both SNV and CNV counts were identified as independent predictors of progression-free survival while NLR, visceral metastasis, and Eastern Cooperative Oncology Group performance status (ECOG PS) (≥1 versus 0) were independent predictors of overall survival.

First of all, the authors^7^ should be commended for their important hypothesis-generating study. It indicates that ICI response prediction is feasible without extensive comprehensive high-throughput analyses by using panel sequencing data and clinical parameters. Of course, as also acknowledged by the authors, critical validation of the suggested biomarkers as well as of the constructed prediction model is warranted, as should be standard for biomarker discovery in general.^9^ In particular, due to the retrospective character of this study, the included patients were treated at different disease stages with different ICI regimens and the panel used for targeted sequencing evolved during the study period. Further, the sequencing panel may have not covered all relevant genes and as the lack of germ-line DNA precluded variant subtraction, benign germline variants might have been considered relevant. These factors may have biased the results of this study.^7^

Another relevant finding of this study^7^ is that in the era/age of “big data” on sophisticated molecular parameters detected in cancer tissues the importance of basic clinical parameters should not be ignored. The clinical parameters identified here are hypothesis-generating as well, as they may point at “host factors” influencing immune responses. These host factors could be of special relevance with respect to the mode of action in immuno-oncological therapies or as surrogates for tumour-specific factors not covered by the biomarker study. In this context, NLR may be a parameter indicating the general activity of the immune system while the site of metastases (visceral versus non-visceral) may reflect differences in tumour biology between individual metastases (lymph node versus hepatic) or between metastases and primary tumour. Especially the latter finding highlights a relevant problem in current studies on biomarker discovery. Biomarker studies are usually performed on primary tumour tissue, as in this study.^7^ However, as in other malignancies, biological parameters of UCs may differ between primary lesions and metastases and may be altered significantly by systemic therapies.^10,11^ The authors do address this concern arguing that analysing the primary untreated lesion instead of new metastatic lesions “reflects common practice”. While this is certainly true currently, in my opinion it should not remain “common practice”. Especially for the discovery of biomarkers for treatment response, the analysed tumour tissue should be as representative as possible of the actual conditions at the start of therapy. This will only be achieved by analysing fresh biopsies, preferably from different metastases. Although this raises ethical issues, we need to discuss whether this is imperative from a scientific point of view in future biomarker studies if we truly wish to answer the demands of precision oncology: who to treat, how to treat, what to treat, when and when not to treat.^12^

## Data Availability

Not applicable
